# Vascularized fibula flap in the management of segmental bone loss following osteomyelitis in children at a Ugandan hospital

**DOI:** 10.5194/jbji-6-179-2021

**Published:** 2021-05-25

**Authors:** Antonio Loro, Andrew Hodges, George William Galiwango, Francesca Loro

**Affiliations:** 1 Orthopedic Department, CoRSU Rehabilitation Hospital, Kisubi, Uganda; 2 Plastic Surgery Department, CoRSU Rehabilitation Hospital, Kisubi, Uganda; 3 Trauma and Orthopedic Department, Bristol Royal Infirmary, Bristol, United Kingdom

## Abstract

**Background**: Hematogenous osteomyelitis is commonly observed in the pediatric population across sub-Saharan Africa. This retrospective case series was designed to evaluate the complications and outcomes of treatment using a vascularized fibula flap (VFF) to fill segmental bone defects secondary to osteomyelitis in children in a low-resource setting in CoRSU Rehabilitation Hospital, Uganda. **Methods**: Clinical notes and radiographs of children with a diagnosis of osteomyelitis that subsequently underwent a VFF procedure between October 2013 and December 2017 were reviewed. All patients were clinically and radiographically evaluated in 2019. **Results**: Forty-four children, with an average bone defect of 10.5 cm, were included. Eighty-four percent of children had successful VFF limb reconstruction. Integration of the graft was radiologically sound in 20.8 weeks on average. The postoperative phase was uneventful in 29 % of patients. Complications were observed in the remaining patients, including flap failure (6), donor leg neurapraxia (3), cutaneous paddle necrosis (11), graft fracture (2), skin graft loss (6), fixator failure (1) and non-union (2). Functional outcomes were rated as excellent in 13 patients, good in 14, fair in 9 and poor in 8. There was no recurrence of the bone infection in any of the enrolled children. **Conclusion**: Despite being a complex and demanding procedure, VFF is a good option for reconstructing post-osteomyelitis bone defects, particularly when associated with loss of soft tissue envelope. Considering the more than satisfactory functional and clinical outcomes, this procedure should be kept in mind for these complex pediatric cases of bone and soft tissue loss, even in a low-resource setting.

## 1 Introduction

Across sub-Saharan Africa, hematogenous osteomyelitis is commonly observed in the pediatric population. Its incidence is unknown, but several papers report that it is an important cause for admission in pediatric units in The Gambia (Bickler and Sanno-Duanda, 2000), Côte D'Ivoire (Kouamé et al., 2005) and Nigeria (Meier et al., 1993). Reports from hospitals located in West Africa suggest that children admitted for osteomyelitis may comprise between 7 %–20 % of all admissions at any given time (Nacoulma et al., 2007; Tekou et al., 2000). This study was conducted at CoRSU Rehabilitation Hospital, Uganda, where, over the last 10 years, the management of osteomyelitis has accounted for 17 %–19 % of all surgical procedures.

In our setting, hematogenous osteomyelitis is usually seen in its chronic stage, which is often with established complications at the time of presentation. One of the most challenging problems is segmental bone loss, particularly when it is extensive and associated with loss of soft tissue cover or destruction of the adjacent joint (in the event of osteomyelitis-septic arthritis). Over the last 10 years, bone defects in our institution have been managed with the following procedures: autogenous grafts (fibular segments or cortical tibial strut), bone transport and vascularized fibula flap (VFF). Of note is the absence of a tissue bank in our setting.

In the literature, there are papers reporting on the use of VFF in the management of bone defects secondary to tumor resection, trauma and post-surgical infection. It is also used in mandibular reconstruction (Peled et al., 2005; Bähr et al., 1998), pseudoarthrosis of the tibia in neurofibromatosis (Daoud and Saighi-Bouaouina, 1989) and avascular necrosis of the femoral head (Fang et al., 2013; Aldridge and Urbaniak, 2007). To our knowledge, the only paper reporting exclusively on the use of VFF in the management of long-bone defects following hematogenous osteomyelitis in children was published by Zalavras et al. (2007); they rated it as a viable option in a case series of eight children. Other publications report case series of post-osteomyelitis defect reconstruction in children but use different operative techniques than VFF (Yeargan et al., 2004; Zahiri et al., 1997; Fowles et al., 1979). Several other papers report results following VFF limb reconstruction; however, in their series, there is a mix of aetiology, age and site (Sun et al., 2010; Arai et al., 2002; Minami et al., 2000; Tang, 1992). Hematogenous osteomyelitis is scarcely represented in their groups, and pediatric patients are small in number. Furthermore, no study on this topic has been performed in sub-Saharan Africa.

In our unit, VFF was included as an option in the treatment of segmental bone loss following osteomyelitis in children in 2013. This retrospective case series was designed to evaluate VFF as a technique for complex limb reconstruction, specifically looking at bone defects secondary to hematogenous osteomyelitis in children. We describe our institution's experience, evaluate postoperative complications and assess the clinical and functional outcomes of this procedure.

### Study site

This study was carried out in CoRSU Rehabilitation Hospital, which is a private not-for-profit facility established in 2009 with the specific mandate to offer orthopedic and plastic services to the pediatric population in Uganda. The team performing VFF consists of seven orthopedic and four plastic surgeons, all full-time employees. The two leading consultant plastic surgeons (Andrew Hodges and George William Galiwango) each have over 10 years of experience in microvascular surgery. During the study period, 103 VFF procedures have been performed to manage osteomyelitis complications, mandibular disorders, congenital pseudoarthrosis of the tibia, and, less frequently, tumor and post-traumatic bone loss. It is the only institution in the region where the procedure is performed routinely.

## 2 Methods

All of the patients who underwent a VFF procedure between October 2013 and December 2017 in our institution were screened for inclusion, as per the following criteria:

- age at surgery ranging from 0 to 15 years;
- diagnosis of osteomyelitis, confirmed by histological report on tissue samples routinely taken intraoperatively at the first surgery;
- underwent a VFF procedure, irrespective of the type (osseous, osteocutaneous or proximal fibular epiphysis).

**Figure 1:**
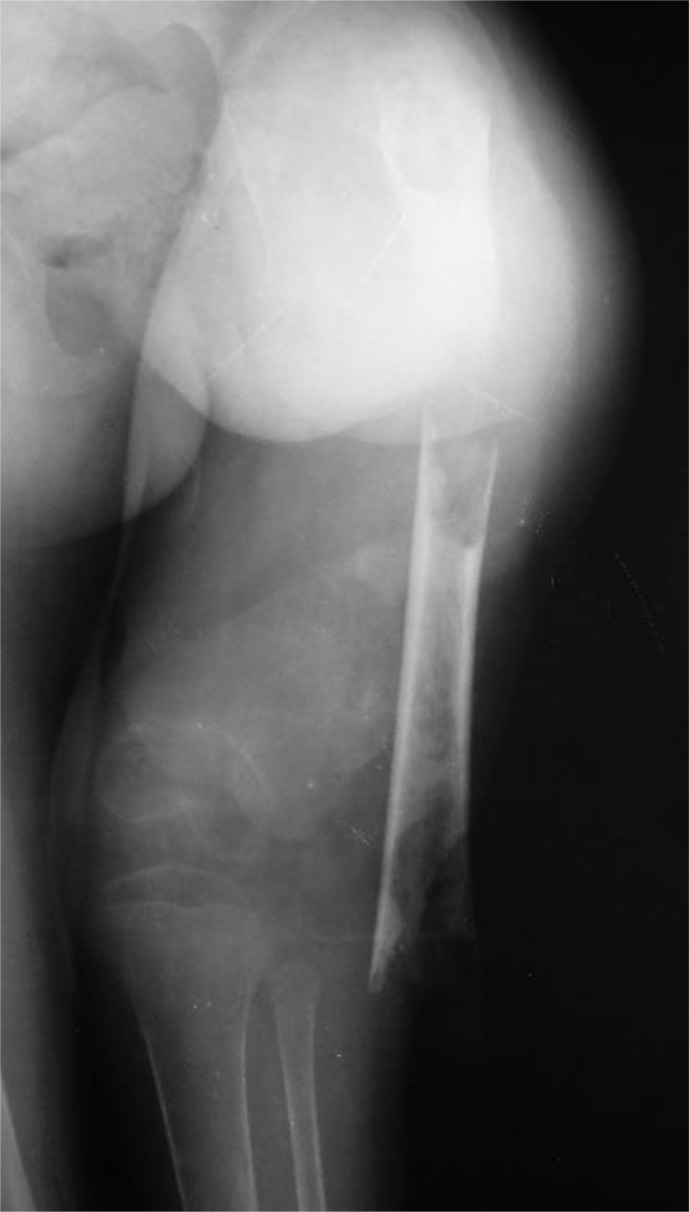
Radiograph of a 5-year-old child who presented with an extruded segment of the femoral diaphysis, skin loss and a flail limb. The short, osteopenic bone stumps do not allow reconstruction that requires good purchase of pins or wires, such as bone transport.

**Figure 2:**
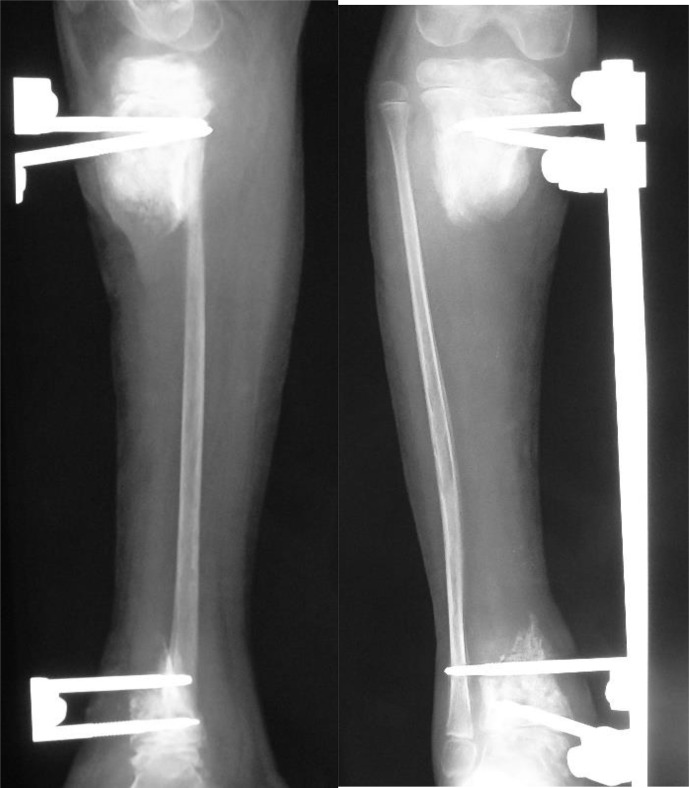
Radiographs showing the post-sequestrectomy bone defect in a 7-year-old child, who presented with exposed sequestrum, loss of soft tissue cover and septic arthritis of the ankle joint. In the presence of short residual stumps and unhealthy skin, the osteocutaneous flap is certainly a valid option for managing both issues.

**Figure 3:**
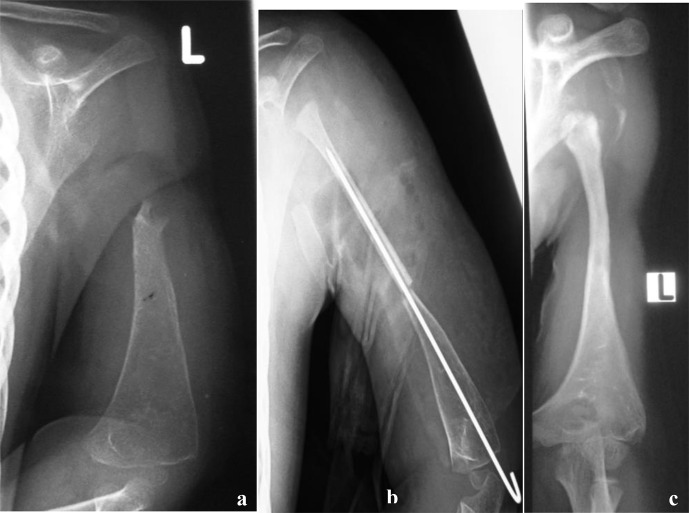
**(a)** Radiograph at presentation of a 3-year-old child referred for a possible shoulder disarticulation; **(b)** a proximal fibular epiphysis was used for bone and joint reconstruction; **(c)** radiograph taken 6 years later demonstrates reconstruction of the proximal half of the humerus, with an expanded and more spherical pseudo-humeral head.

VFF was indicated as a procedure for complex cases in which both of the following conditions were met and therefore precluding the use of other reconstructive techniques:

- presence of a post-osteomyelitis segmental bone defect exceeding 5 cm, which is isolated or associated with loss, or poor status, of local soft tissue (Fig. 1);
- presence of either
  •: short residual bone stumps (Fig. 2) or
  •: bone defect associated with destruction of one of the adjacent joints (Fig. 3).

Children presenting with post-traumatic osteomyelitis were excluded in order to have a diagnostically homogeneous series. All of the included participants were assessed, examined and underwent radiographic imaging in 2019 during a follow-up visit.

Treatment was considered successful at their last follow-up visit if infection control, reconstruction of bone continuity and revival of soft tissues were all achieved. Children and/or their families were asked to subjectively rate their own functional outcomes, based on their ability to perform the activities of daily living (ADLs) that are expected from the children in their social context (see Table 6 for definitions).

### 2.1 Management prior to VFF

Prior to reconstruction, each child underwent surgeries indicated for the eradication of the infection and/or optimization of the recipient bed. In the presence of an active infection, radical debridement was performed in the first instance, with removal of all necrotic tissue. However, in cases presenting without a clear extent of sequestration, the radical debridement was preceded by stabilization of the bone with external fixation in order to achieve a clearer demarcation of the sequestered segment and, possibly, a better involucrum. Stabilization in the pre-reconstruction stage can lead to resizing of the sequestrum, decreasing the length of the resulting bone defect once the definitive debridement is carried out. In quiescent cases, external fixation was utilized for preoperative axis correction and lengthening of the segment. The fixator was usually left in place until the reconstruction stage; adjustments were performed in case of screws loosening due to pin tract infection.

In cases of soft tissue loss, the wound was left open to granulate or, when required, vacuum assisted closure (VAC) was utilized. Intraoperative biopsy was routinely done to confirm the diagnosis and to rule out tuberculosis. Antibiotic beads, cement spacers or limb arteriography was not used in our series.

### 2.2 Vascular fibula flap operative procedure

The VFF procedure was performed once clinical signs (lack of drainage, swelling and pain in the recipient site and good nutritional status), laboratory values (hemoglobin >10 g/dL, blood slide negative for malaria parasites) and radiographic findings (no sequestra seen on recent radiographs) suggested eradication of infection. The surgical team included at least two plastic surgeons, an orthopaedic surgeon and a pediatric anesthesiologist. The standard procedure for raising the fibula was used, preserving both knee and ankle stability (Graham et al., 2003; Masquelet and Gilbert, 2001; Wei et al., 1986; Taylor et al., 1975). No screw fixation was done to stabilize the distal fragment of the fibula. All dissections were performed with loupe magnification (3.5× and 4.0×) and, when possible, with a microscope for the recipient vessels. All anastomoses were performed under the microscope (Zeiss OPMI S88). A basic set for hand and reconstructive microsurgery was used. Flaps were stabilized by either internal or external fixation and sometimes in combination (Table 3). When required and if available, intraoperative fluoroscopy was utilized for pin and wire placement. A handheld Doppler scanner with 8 Hz probe was used to assess the donor and recipient vessels preoperatively and the flap pedicle for the first 36 h postoperatively. Monitoring of an osseous flap was done with the same technique: buried flap pedicles were not monitored by Doppler in our series.

## 3 Results

### 3.1 Cohort and operation characteristics

Forty-four children were included in this study. Forty-five VFF procedures were done; one child underwent a second VFF at the same site after failure of the first. Forty-three were followed up, both clinically and radiologically, for an average period of 29.2 months (range 15–70 months). One patient was lost to follow-up after the initial admission. The cohort characteristics are summarized in Table 1.

**Table 1:** Cohort characteristics.

Patient characteristics	n	%
Sex		
Male	28	64
Female	16	36
Age (years)		
0–5	13	29
6–10	21	48
11–15	10	23
Location of bone defects${}^{*}$		
Tibia	27	60
Femur	8	18
Radius	6	13
Humerus	4	9
Associated conditions at presentation		
Pathological fractures (all spontaneous)	17	39
Angular deformities	10	22
Limb length discrepancy (LLD)	14	32
Loss of entire bone	2	5
Destruction of adjacent joint	3	7
Exposed sequestra with skin loss	19	43
Secondary site of osteomyelitis	4	9

${}^{*}$ out of 45 procedures

The average age at presentation was 7.6 years (range 2–14 years). The mean duration of infection at presentation was 8.7 months (range 1–48 months). Eighty-four percent of children presented with active osteomyelitis on admission; 16 % of the children were referred for limb reconstruction following surgery performed in other institutions.

Ability to use the limb was greatly reduced in more than 60 % of the children; furthermore, 7 % of children showed impaired function of an adjacent joint. Sixteen children had a moderate anaemia with hemoglobin <10 g/dL on admission; two of them had hemoglobin <7.5 g/dL requiring red blood cell transfusion.

Prior to reconstruction, each child underwent an average of two surgeries (range one to seven interventions), summarized in Table 2. The time to prepare the limb for VFF was 0–90 d in 36 % of patients, 91–180 d in 41 % of patients, and more than 180 d in 23 % of patients.

**Table 2:** Procedures performed before VFF.

Surgeries before VFF	n
Sequestrectomy	24
Sequestrectomy + external fixation	27
External fixation to allow sequestrum demarcation	8
External fixation for pre-op. distraction	8
External fixation + fibula osteotomy	2
External fixation + incision and drainage	1
Incision and drainage	8
Screw replacement and fixator adjustment	10
Bone graft	2

The average operative time for the VFF procedure was 6.5 h (range 4.5–10.5 h). On average, the length of the skeletal defect was 10.5 cm (range 6–20 cm), and the length of the fibula graft was 13.6 cm (range 7–23 cm). Types and methods of flap fixation are listed in Table 3. External fixators were used in 36 patients, most commonly in lower-limb reconstruction.

**Table 3:** VFF procedure characteristics.

Variable	n (N=45)	%
Type of fibula harvest		
Osseous flap: shaft segment	6	13
Osteocutaneous flap: shaft segment with skin island/paddle	34	76
Proximal fibular flap: shaft segment with proximal epiphysis (with or without skin paddle)	5	11
Method of flap fixation		
Kirschner's wire	5	11.1
Kirschner's wire plus external fixation	3	6.7
Kirschner's wire plus cast	1	2.2
External fixation	26	57.7
Plate plus external fixation	1	2.2
Cast	1	2.2
Plate	1	2.2
External fixation plus wire cerclage	1	2.2
External fixation plus screw	5	11.1
Rush rod	1	2.2

### 3.2 Complications

The postoperative phase was uneventful in 29 % of cases. Complications were noted in the remaining 71 %, both in the donor and recipient sites (Tables 4 and 5, respectively).

**Table 4:** Donor site complications following VFF.

Donor site complications	n
Infection	4
Neurapraxia${}^{*}$	3
Skin graft loss	6

${}^{*}$ All foot drops were managed with temporary splint and fully recovered within 4 months.

**Table 5:** Recipient site complications following VFF.

Recipient site complications	n
Flap failure	5
Flap resorption	1
Vascular disturbances	7${}^{*}$
Loss of skin graft	6
Skin paddle necrosis	11
Graft mal-alignment	3
Graft fracture	2
Graft infection	5
Graft delayed union	2
Graft non-union	2
Fixator failure	1

${}^{*}$ The osteocutaneous flap failed in two cases and had to be completely removed; the other five patients underwent further surgeries and the flaps survived.

Of the 74 flap-recipient bone junctions, only 2 cases of non-union and 2 cases of delayed union were observed (5 %); none of them were infected. Niches of infection in an already osteointegrated graft were experienced in five cases, mostly sequelae of infected pin tracks.

Deformities were noted in 31 patients who underwent lower-limb reconstruction. Of the 27 tibial reconstructions, 10 cases of limb length discrepancy (LLD) (range 2–14 cm) and 7 cases of fused ankles were noted. Foot deformity and axial deformities were observed in five patients. All seven cases of femur reconstruction demonstrated LLD (range 6–16 cm) and a stiff knee. Notably, LLD, axial deformity, and knee and ankle joint arthritis were present in 31 out of 34 patients before reconstruction. In the upper limb, deformities were seen in three out of six forearm procedures (radial club hand and deformed wrist) and in two out of four humeral reconstructions (shortening and retracting scars).

### 3.3 Outcomes

Successful treatment was achieved in 38 children (84 %). In six (16 %), the procedure was unsuccessful. Of these, five failed due to flap related issues (two flap failures secondary to thrombosis and three flap failures secondary to infection) and one graft (to the radius) failed due to complete graft resorption.

**Figure 4:**
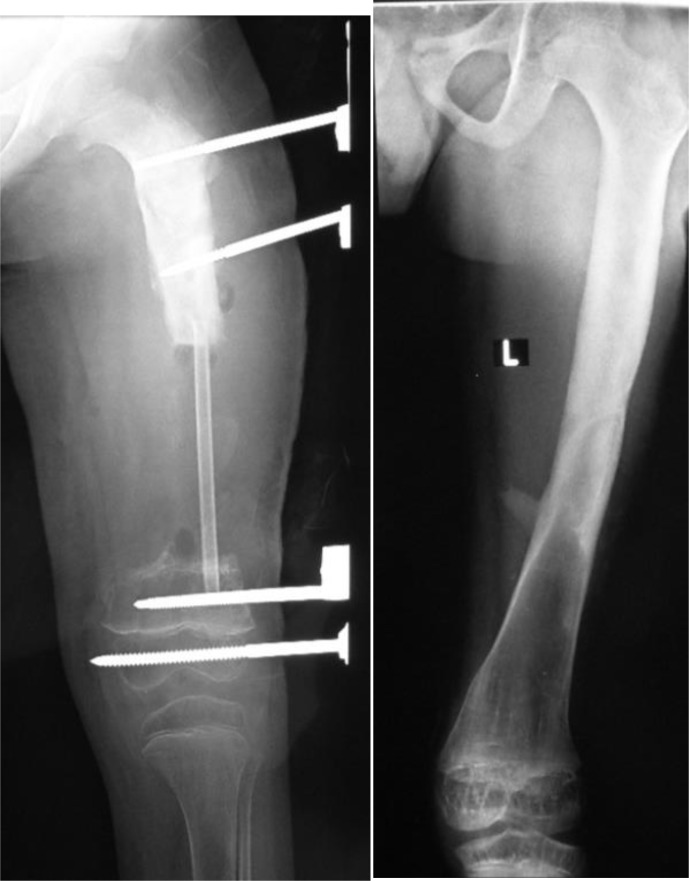
Radiographs taken 5 years apart following an osseous graft in the femur of a 5-year-old boy. Remodeling has made the grafted segment indistinguishable from the host bone.

On average integration of the graft was radiologically sound in 20.8 weeks (range 8–104 weeks). In the upper limb it required 11.8 weeks as compared to 24 weeks in the lower limb. All but two grafts underwent marked remodeling and hypertrophy (Fig. 4). Full weight-bearing was allowed 6 months after the index procedure (range 4–13 months).

Functional outcomes are reported in Table 6, adapting the criteria reported by Tang (1992) to our cohort and social context. At the last follow-up visit, further surgery was deemed necessary in 31 children in order to improve gait pattern and limb usage. The required procedures will include joint fusion, axis correction or bone lengthening.

**Table 6:** Functional outcomes, as rated by the patients, at the last follow-up visit.

Functional outcome rating	Definition	N	%
Excellent	ADL${}^{*}$ performed without restrictions and without pain	13	29.5
Good	ADL performed with minimal difficulty with or without mild pain in reconstructed limb	14	31.8
Fair	ADL performed with difficulty with or without mild pain in the reconstructed limb	9	20.5
Poor	ADL performed with significant limitations with or without moderate-to-severe pain in the reconstructed limb. Need to use walking aids or orthotic devices due to limb shortening or deformities	8	18.2

${}^{*}$ ADL (activities of daily living). Examples of ADLs for upper limb are the following: able to perform tasks at school, able to wash clothes, able to carry jerrycan for water, and able to peel vegetables and cook. Examples of ADLs for lower limb are the following: able to squat, able to run and walk normal distances expected for patient's age, able to fetch water or firewood, and able to dig.

## 4 Discussion

In managing a bone defect, the surgeon's choice depends not only on multiple clinical and radiological factors but also on their experience and working environment (Rasool, 2011).

**Figure 5:**
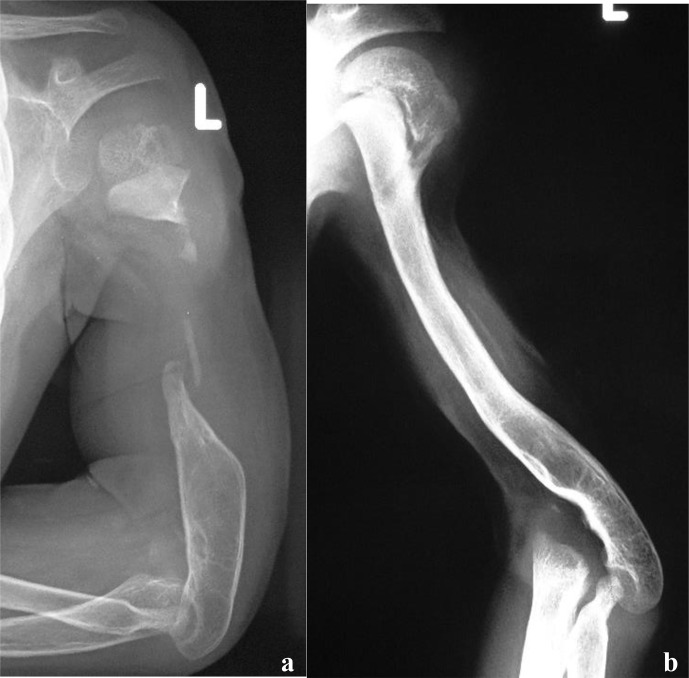
**(a)** Radiographs, at presentation, of a 2-year-old boy who presented with bone loss and sequelae of septic arthritis of the elbow joint. There was a history of two previous attempts to fill the gap with cortico-cancellous grafts. Reconstruction options were limited by the short stumps, severe osteopenia and the previous clinical history. **(b)** Radiographs taken at last follow-up, 4 years after VFF; soundly integrated graft with excellent functional results.

**Figure 6:**
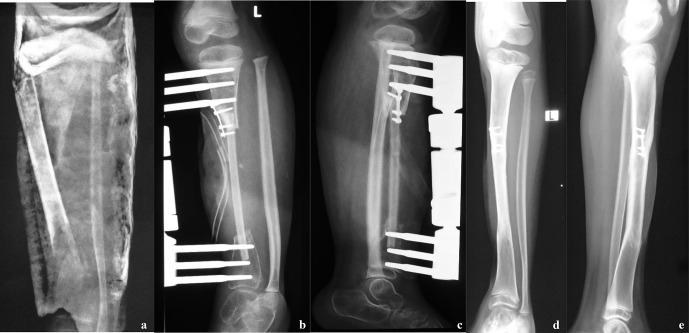
**(a)** Radiographs taken at presentation of a 5-year-old girl with an extruding sequestrum of the left tibia, with soft tissue loss; **(b, c)** post-VFF radiographs; **(d, e)** radiographs taken 3 years postoperatively. There is sound bone reconstruction, with perfect integration of the VFF, hypertrophy and remodeling.

Rasool (2008) gives an overview of the different techniques for limb reconstruction, with specific attention to their use in developing countries. In many of our cases, however, we found it difficult to employ these techniques due to the extent of the bony defect, the condition of the soft tissues, the length of the remaining epiphyseal–metaphyseal bone stumps or the destroyed adjacent joints. In 2013, we hypothesized that VFF presented a suitable option to overcome these challenges. The vascularized fibula has important anatomical and biomechanical characteristics: it is mechanically strong with a dual vascular supply, it is more resistant to infections when compared to the non-vascularized one (Goldberg et al., 1987), and, in children, the size matches that of the radius, humerus, and tibia. Furthermore, it can be harvested according to reconstructive requirements. The grafted fibula, once soundly integrated and hypertrophied, can also be lengthened by employing the standard procedures. All of these characteristics allow the surgeon to perform an individualized child-tailored treatment. The radiological sequences shown in Figs. 5 and 6 demonstrate the inherent plasticity of the fibula graft over time.

The strengths of this study are its diagnostic homogeneity, its cohort size and its setting in an as yet unstudied population in sub-Saharan Africa. This study is a retrospective case series, with the limitations of this study type. It evaluates VFF as a procedure indicated specifically for challenging cases for which other reconstructive procedures were not possible or not feasible. Unfortunately, it could not therefore be directly compared to a gold standard.

The financial costs represent an important aspect, especially for fragile health systems and strict budgets. All the surgeries were subsidized or paid for by good Samaritans, charities and hospital management. This has been, and most probably will be, the only way to continue carrying out these limb salvage procedures in low-income countries in the coming years.

### 4.1 Choice of graft

The choice of flap was dictated by the reconstructive needs, the degree of scarring and the vascularity of the recipient site. In the event of a healthy soft tissue envelope or in the presence of a good muscular cushion, as for femur and humerus, the osseous flap was preferred. When combined reconstruction of bone and soft tissues was required, the osteocutaneous flap was utilized, mostly in the tibia and less frequently in the radius. The skin paddle allows the surgeon the freedom to excise all scarred and poorly vascularized overlying skin. This in turn allows for a thorough debridement of the recipient bed. The risk of post-surgical skin necrosis, associated with reconstructive options requiring open access to the recipient graft site, is lowered. For this reason, the use of a fibular transfer is advised in cases of significant scarring of the leg and in a poorly vascularized bed (Rasool, 2008). The proximal fibular epiphyseal graft was chosen for the reconstruction of defects of the distal half of the radius and the proximal end of the humerus. Innocenti et al. (2004) reported the harvesting technique and the use of this type of graft in six children with bone defects following tumor resection of the distal radius. In one case in our series, it was used to reconstruct the upper third of the femur and its neck–head complex; unfortunately, the flap became infected and had to be removed.

### 4.2 Complications

Successful microvascular reconstruction rates are recognized to be lower in chronic wounds (Rasool, 2011; Organek et al., 2006; Vail and Urbaniak, 1996; Daoud and Saighi-Bouaouina, 1989) and in low-resource settings, even if performed by experienced teams (Huijing et al., 2011; Marck et al., 2010). A review of postoperative outcomes and challenges associated with free flaps in our institution was published by Citron et al. (2016). Although this review is not limited to VFF, it analyses free flap procedures carried out by the same plastic surgeons as in this case series. It states that surgical expertise, late and complex presentations, limited resources, and postoperative care challenges are all important contributing factors to outcomes in our institution. This review also evaluated the learning curve associated with these procedures in our setting and found a significant improvement in outcomes over time (Citron et al., 2016).

In this case series, there were seven vascular complications. These cases were all rated as difficult by our plastic surgeons since the scarred and fibrotic tissues made dissection of the recipient vessels very arduous. In addition, in children, the vessels are more prone to spasm, particularly if a small branch is missed. Meticulous dissection under good magnification is required in order to ensure ligation of all branches. Furthermore, the diameter of children's vessels is small (2–4 mm), and this makes it more challenging for end-to-side anastomosis; in our case series, therefore, end-to-end anastomosis was used preferentially. Minami et al. (2000) and Zalavras et al. (2007) reported similar percentages of vascular complications in their studies, with 13.5 % and 12.5 %, respectively. Zahiri et al. (1997) reported no vascular complications in his series of nine children.

Great attention should be given to the skin paddle since superficial (epidermolysis) and partial skin flap necrosis may occur. This is a complication reported by several authors. Minami et al. (2000) had three cases of flap necrosis out of nine, while Arai et al. (2002) had eight cases out of nine following venous congestion. Additional procedures may be necessary in order to save the perfusion and to avoid exposure of the graft. In our cases, skin paddle survival in struggling flaps required additional surgical procedures to improve perfusion, especially in the first 10 postoperative days. In one case, the flap required seven additional surgeries. In two other cases, local fasciocutaneous flaps were required for partial skin paddle loss resulting in partial exposure of the grafted fibula. Interestingly, a delayed union of the graft was noted at 1 year from surgery in both cases.

In our series, external fixators were very commonly used. Two cases of knee varus deformity and two cases of ankle fusion seen in our case series might be attributable to either the positioning errors of the screws causing damage to the growth plate or to the early closure of these plates following septic loosening of the screws. Hence, if available, we strongly recommend the use of intraoperative fluoroscopy in challenging cases. Furthermore, great emphasis should also to be put on fixator care, in order to try to minimize pin tract infections.

Donor site morbidity following the harvest of the fibula is well documented (Verma et al., 2016; Agarwal et al., 2012; Babovic et al., 2000; Shpitzer et al., 1997; Coghlan and Townsend, 1993). A systematic review revealed that up to 10 % of the patients may subsequently develop ankle pain, instability and/or valgus deformity, chronic leg pain, cold intolerance, and motor weakness (Ling and Peng, 2012). Sensory impairments have also been reported in several papers (Verma et al., 2016; Ling and Peng, 2012; Babovic et al., 2000; Shpitzer et al., 1997; Vail and Urbaniak, 1996).

## 5 Conclusion

This retrospective case series has shown that the VFF procedure is a valid option in reconstructing challenging post-osteomyelitis bone defects in children in low-income countries. It shows that VFF may lead to successful management of very complex problems by controlling the infection and by recovering limb function in a good number of cases. Postoperative complications are multiple and common, and each child is likely to need multiple surgeries. This technique could be adopted, under the right conditions, in other institutions located in low-income countries, although further prospective studies are needed.

## Data Availability

The data that support the findings of this study are openly available in figshare at (Loro and Loro, 2021).

## References

[bib1.bib1] Agarwal DK, Saseendar S, Patro DK, Menon J (2012). Outcomes and complications of fibular head resection. Strategies Trauma Limb Reconstr.

[bib1.bib2] Aldridge III JM, Urbaniak JR (2007). Avascular necrosis of the femoral head: role of vascularized bone grafts. Orthop Clin N A.

[bib1.bib3] Arai K, Toh S, Tsubo K, Nishikawa S, Narita S, Miura H (2002). Complications of vascularized fibula graft for reconstruction of long bones. Plast Reconstr Surg.

[bib1.bib4] Babovic S, Johnson CH, Finical SJ (2000). Free fibula donor-site morbidity: the Mayo experience with 100 consecutive harvests. J Reconstr Microsurg.

[bib1.bib5] Bähr W, Stoll P, Wächter R (1998). Use of the “double barrel” free vascularized fibula in mandibular reconstruction. J Oral Maxillofac Surg.

[bib1.bib6] Bickler SW, Sanno-Duanda B (2000). Epidemiology of paediatric surgical admissions to a government referral hospital in the Gambia. Bull World Health Organ.

[bib1.bib7] Citron I, Galiwango G, Hodges A (2016). Challenges in global microsurgery: A six year review of outcomes at an East African hospital. J Plast Reconstr Aesthet Surg.

[bib1.bib8] Coghlan BA, Townsend PLG (1993). The morbidity of the free vascularised fibula flap. Brit J Plast Surg.

[bib1.bib9] Daoud A, Saighi-Bouaouina A (1989). Treatment of sequestra, pseudarthroses, and defects in the long bones of children who have chronic hematogenous osteomyelitis. J Bone Joint Surg Am.

[bib1.bib10] Fang T, Zhang EW, Sailes FC, McGuire RA, Lineaweaver WC, Zhang F (2013). Vascularized fibular grafts in patients with avascular necrosis of femoral head: a systematic review and meta-analysis. Arch Orthop Trauma Surg.

[bib1.bib11] Fowles JV, Lehoux J, Zlitni M, Kassab MT, Nolan B (1979). Tibial defect due to acute haematogenous osteomyelitis: treatment and results in twenty-one children. J Bone Joint Surg Br.

[bib1.bib12] Goldberg VM, Shaffer JW, Field G, Davy DT (1987). Biology of vascularized bone grafts. Orthop Clin N AM.

[bib1.bib13] Graham RG, Swan MC, Hudson DA, van Zyl JE (2003). The fibula free flap: advantages of the muscle sparing technique. Br J Plast Surg.

[bib1.bib14] Huijing MA, Marck KW, Combes J, Mizen KD, Fourie L, Demisse Y, Befikadu S, McGurk M (2011). Facial reconstruction in the developing world: a complicated matter. Br J Oral Maxillofac Surg.

[bib1.bib15] Innocenti M, Delcroix L, Manfrini M, Ceruso M, Capanna R (2004). Vascularized proximal fibular epiphyseal transfer for distal radial reconstruction. J Bone Joint Surg Am.

[bib1.bib16] Kouamé BD, Dick KR, Ouattara O, Gouli JC, Odéhouri THK, Coulibaly C (2005). Traitement des ostéomyélites compliquées de l'enfant au CHU de Yopougon, Abidjan (Côte d'Ivoire). Cahiers d'études et de recherches francophones/Santé.

[bib1.bib17] Ling XF, Peng X (2012). What is the price to pay for a free fibula flap? A systematic review of donor-site morbidity following free fibula flap surgery. Plast Reconstr Surg.

[bib1.bib18] Loro F, Loro A (2021). figshare.

[bib1.bib19] Marck R, Huijing M, Vest D, Eshete M, Marck K, McGurk M (2010). Early outcome of facial reconstructive surgery abroad: a comparative study. Eur J Plast Surg.

[bib1.bib20] Masquelet AC, Gilbert A (2001). An atlas of flaps of the musculoskeletal system.

[bib1.bib21] Meier DE, Tarpley J, Olaolorun DA, Howard CR, Price CT (1993). Hematogenous osteomyelitis in the developing world: a practical approach to classification and
treatment with limited resources. Contemporary Orthopaedics.

[bib1.bib22] Minami A, Kasashima T, Iwasaki N, Kato H, Kaneda K (2000). Vascularised fibular grafts. An experience of 102 patients. J Bone Joint Surg Br.

[bib1.bib23] Nacoulma S, Ouedraogo D, Nacoulma E, Korsaga A, Drabo J (2007). Ostéomyélites chroniques au CHU de Ouagadougou (Burkina Faso). Étude rétrospective de 102 cas (1996–2000). B Soc Pathol Exot.

[bib1.bib24] Organek AJ, Klebuc MJ, Zuker RM (2006). Indications and outcomes of free tissue transfer to the lower extremity in children: review. J Reconstr Microsurg.

[bib1.bib25] Peled M, El-Naaj IA, Lipin Y, Ardekian L (2005). The use of free fibular flap for functional mandibular reconstruction. J Oral Maxillofac Surg.

[bib1.bib26] Rasool MN (2008). The treatment of tibial defects following chronic pyogenic haematogenous osteomyelitis in children. South African Orthopaedic Journal.

[bib1.bib27] Rasool MN (2011). Pyogenic osteomyelitis of the forearm bones in children. South African Orthopaedic Journal.

[bib1.bib28] Shpitzer T, Neligan P, Boyd B, Gullane P, Gur E, Freeman J (1997). Leg morbidity and function following fibular free flap harvest. Ann Plast Surg.

[bib1.bib29] Sun Y, Zhang C, Jin D, Sheng J, Cheng X, Liu X, Chen S, Zeng B (2010). Free vascularised fibular grafting in the treatment of large skeletal defects due to osteomyelitis. Int Orthop.

[bib1.bib30] Tang CH (1992). Reconstruction of the bones and joints of the upper extremity by vascularized free fibular graft: report of 46 cases. J Reconstr Microsurg.

[bib1.bib31] Taylor GI, Miller GD, Ham FJ (1975). The free vascularized bone graft. A clinical extension of microvascular techniques. Plast Reconstr Surg.

[bib1.bib32] Tekou H, Foly A, Akue B (2000). Le profil actuel des ostéomyelites hématogènes de l'enfant au centre hospitalier universitaire de Tokoin, Lomé, Togo, à propos de 145 cas. Médecine tropicale.

[bib1.bib33] Vail TP, Urbaniak JR (1996). Donor-site morbidity with use of vascularized autogenous fibular grafts. J Bone Joint Surg Am.

[bib1.bib34] Verma AK, Kushwaha NS, Saini A, Waliullah S, Navadaya MK, Kumar D (2016). Retrospective Analysis of Donor Site Morbidity Following Partial Fibular Resection. International Journal of Contemporary Medical Research.

[bib1.bib35] Wei FC, Chen HC, Chuang CC, Noordhoff MS (1986). Fibular osteoseptocutaneous flap: anatomic study and clinical application. Plast Reconstr Surg.

[bib1.bib36] Yeargan 3rd SA, Nakasone CK, Shaieb MD, Montgomery WP, Reinker KA (2004). Treatment of chronic osteomyelitis in children resistant to previous therapy. J Pediatr Orthop.

[bib1.bib37] Zahiri C, Zahiri H, Tehrany F (1997). Limb salvage in advanced chronic osteomyelitis in children. Int Orthop.

[bib1.bib38] Zalavras CG, Femino D, Triche R, Zionts L, Stevanovic M (2007). Reconstruction of large skeletal defects due to osteomyelitis with the vascularized fibular graft in children. J Bone Joint Surg Am.

